# Evaluation of the Effectiveness of MicroMega Remover, ProTaper Universal Retreatment, Reciproc, and Hedstrom Files in the Retreatment of Curved Root Canals Obturated with Different Techniques: A Micro-Computed Tomography Study

**DOI:** 10.3390/medicina62010188

**Published:** 2026-01-16

**Authors:** Pınar Hava Dursun, Fatma Semra Sevimay, Arda Buyuksungur, Berkan Celikten

**Affiliations:** 1 Department of Endodontics, Faculty of Dentistry, Ankara University, Ankara 06560, Turkey; ssevimay@dentistry.ankara.edu.tr (F.S.S.); bcelikten@ankara.edu.tr (B.C.); 2 Department of Basic Medical Sciences, Faculty of Dentistry, Ankara University, Ankara 06560, Turkey; abuyuksungur@ankara.edu.tr

**Keywords:** apical transportation, dental instruments, endodontics, micro-computed tomography, retreatment, root canal obturation

## Abstract

*Background and Objectives*: The anatomically demanding structure of curved root canals increases the technical difficulty of retreatment procedures. This study aimed to evaluate the retreatment efficacy of various rotary and reciprocating instruments in curved root canals obturated with cold and warm techniques regarding root canal filling material removal, apical transportation, and retreatment time. *Materials and Methods*: Sixty-four curved mesial root canals of mandibular molars with Vertucci type IV morphology were prepared using the ProTaper Gold system and obturated with AH Plus sealer using either the single-cone (SC) (n = 32) or continuous wave vertical compaction (CWC) (n = 32) technique. Each group was divided into four subgroups (n = 8) and retreated using MicroMega Remover (MM Remover), ProTaper Universal Retreatment (PTUR), Reciproc (Rec), and Hedstrom file systems. Micro-computed tomography was used to assess residual filling material volume and apical transportation. The time required for retreatment was recorded. The level of statistical significance was set at *p* < 0.05. *Results*: Across both obturation techniques, the MM Remover and PTUR groups demonstrated shorter retreatment times compared with the CWC–Hedstrom group (*p* < 0.05). No statistically significant differences were observed among the file systems in terms of filling material removal and apical transportation (*p* > 0.05). *Conclusions*: All tested instruments effectively preserved root canal anatomy within clinically acceptable apical transportation limits. The MM Remover and PTUR systems achieved significantly shorter retreatment times, indicating clinical advantages in efficiency. None of the evaluated systems achieved complete removal of the filling materials in either obturation technique. This study provides one of the first comparative evaluations of the MM Remover system, supporting its applicability in complex canal configurations.

## 1. Introduction

Root canal (RC) therapy aims to eradicate intraradicular infection, allowing periapical healing and preserving tooth function [1]. Despite high success rates of endodontic therapy (86–98%) [2], failures still occur for various reasons [3]. In such situations, nonsurgical retreatment is commonly considered the preferred initial management approach [1]. An essential phase of nonsurgical retreatment involves removing the previous RC filling material, as remnants can harbor microorganisms and impair the success of disinfection and re-obturation [4]. However, the literature indicates that no technique can completely remove filling materials from the RC system [5,6,7], and achieving complete cleanliness remains a clinical challenge.

Hedstrom hand files (Dentsply, Ballaigues, Switzerland) remain widely used in endodontic retreatment procedures due to their sharp cutting-edge design, which facilitates effective engagement and removal of RC filling materials [8]. Their retreatment performance has been assessed in several previous studies [9,10,11]. Nickel–Titanium (NiTi) file systems are favored for flexibility, high fracture resistance, efficiency, and ease of use [12]. The ProTaper Universal Retreatment (PTUR) system (Dentsply, Ballaigues, Switzerland), a multi-file NiTi instrument specifically developed for retreatment procedures, is commonly used in both curved and straight RCs [6,13,14]. The Reciproc (Rec) system (VDW, Munich, Germany), a single-file reciprocating instrument, has also been shown to effectively remove filling materials during retreatment while maintaining procedural efficiency [5,6,7,15]. The MicroMega Remover (MM Remover) (Micro-Mega, Besancon, France) is a newly developed single-file retreatment instrument manufactured using heat-treated C-wire technology and featuring a variable triple-helix cross-sectional design [16]. According to the manufacturer, the file is designed to mechanically remove gutta-percha without the use of solvents and, owing to its non-cutting tip, aims to minimize procedure-related risks while preserving the RC anatomy [17]. It is recommended for use at a rotational speed of 400–800 rpm with a torque setting of 2.5 Ncm. A recent study reported that the MM Remover demonstrated higher cyclic fatigue resistance than PTUR [16]. However, comprehensive studies assessing the retreatment performance of the MM Remover remain limited.

In recent years, warm obturation techniques have gained widespread acceptance as they provide a more homogeneous and well-adapted gutta-percha mass within complex RC anatomies. Nevertheless, these techniques may increase the difficulty of filling removal during retreatment, especially in severely curved RCs, where risks such as apical transportation, ledge formation, or perforation are elevated [7,18]. Therefore, the selection of appropriate file systems during retreatment is crucial to achieve optimal clinical outcomes.

Tomography-based imaging modalities are widely used in endodontic research due to their ability to provide three-dimensional (3D) visualization of RC systems [19,20]. Micro-computed tomography (micro-CT) provides non-invasive, high-resolution, and reproducible evaluations [7,21]. Owing to these advantages, it has been extensively employed to investigate RC morphology before and after instrumentation, including the assessment of dentin volume alterations [22]. Moreover, micro-CT has been used to evaluate debris removal following RC irrigation, residual filling material, and the shaping ability of endodontic instruments [19,23].

Although various micro-CT studies have evaluated different retreatment systems, comprehensive investigations assessing the performance of newly developed instruments are still required. In this context, the design features of the MM Remover, including a heat-treated C-wire alloy and a variable triple-helix cross-sectional geometry, may influence both the efficiency of filling material removal and apical transportation, particularly in curved RC anatomies, which are clinically challenging during nonsurgical retreatment. Such comparative data obtained under varying experimental conditions are essential for clarifying the clinical applicability of this system. According to the current literature, there is no available micro-CT study comparing the performance of the recently introduced MM Remover with established rotary and reciprocating file systems under different obturation techniques. Therefore, this in vitro study evaluated the effectiveness of four different instruments in terms of RC filling material removal and apical transportation in curved RCs obturated with different techniques using micro-CT. The instruments evaluated included PTUR, Rec, Hedstrom files, and the MM Remover. Additionally, the time required for RC filling material removal was compared.

## 2. Materials and Methods

### 2.1. Ethical Approval and Sample Size Calculation

Ethical approval for this research was obtained from the Ethics Committee of Ankara University Faculty of Dentistry (Decision No. 10/06, dated 19 June 2023). The study protocol was conducted in accordance with the Declaration of Helsinki. Before the teeth were extracted and collected, all participants were fully informed and provided written informed consent. Sample size estimation was conducted using G*Power software (version 3.1.9.7; Dusseldorf, Germany). Based on residual filling material as the primary outcome, an effect size of 0.6, a statistical power of 80%, and a type I error rate of 5% were applied, indicating that a minimum of six specimens per subgroup were required. Adequate power for apical transportation was verified by post-hoc analysis. Accordingly, eight specimens were included per group.

#### Sample Preparation, Inclusion, and Exclusion Criteria

The mesial roots of fully developed permanent mandibular molars extracted for periodontal reasons were included; teeth with restorations, previous RC treatment, fractures, cracks, or resorptions were excluded. Root surfaces were cleaned with periodontal curettes, and the teeth were stored in 0.1% thymol solution. Root curvature was assessed radiographically, and, according to Schneider’s classification [24], teeth with severe mesial curvature (25–40°) were selected. In total, 64 mesial RCs with Vertucci type IV configuration were included [25]. To achieve standardized root lengths of 16 ± 1 mm, the crowns were sectioned and access cavities were prepared with a diamond fissure bur.

### 2.2. Root Canal Preparation and Obturation

Apical patency of mesiobuccal and mesiolingual RCs was verified with a #10 K-file (Dentsply Maillefer, Ballaigues, Switzerland). The working length (WL) was determined at 1 mm coronal to the apical foramen. RCs were prepared to F2 (25/0.06) with the ProTaper Gold (Dentsply Sirona, Ballaigues, Switzerland), driven by an endodontic motor (AI Motor T-mode Yoshi Terauchi Edition; Woodpecker, Guilin, China). Throughout instrumentation, 2.5% sodium hypochlorite (NaOCl) (Microvem, Istanbul, Turkey) was used as the irrigant. The smear layer was removed by sequential irrigation with 17% ethylenediaminetetraacetic acid (EDTA) (Promida, Eskisehir, Turkey), 2.5% NaOCl, and distilled water (2 mL each). Finally, the RCs were dried with sterile paper points.

Samples were randomized to single-cone (SC; Group A) (n = 32/group) or continuous wave vertical compaction (CWC; Group B) (n = 32/group). AH Plus sealer (Dentsply Maillefer, Ballaigues, Switzerland) with F2 gutta-percha was used in both groups. In the SC group, gutta-percha cones were cut at the canal orifice and condensed with a plugger. In the CWC group, the F2 master cone was cut with the Elements Free down-pack device at 200 °C (Kerr Endodontics, Orange, CA, USA), leaving five mm apically, and compacted with a size 1 hand plugger (Machtou plugger; VDW, Munich, Germany). Backfilling at 100 °C was performed in 3–4 mm increments, and gutta-percha was condensed with pluggers (sizes 2–4) until obturation was complete. Coronal access cavities were temporarily sealed with Cavit-G (3M ESPE, Seefeld, Germany). Obturation quality was verified radiographically in buccolingual and mesiodistal projections. Samples were stored at 37 °C in 100% relative humidity for 14 days to allow sealer setting.

### 2.3. Root Canal Retreatment

The obturated samples were randomly allocated to four retreatment subgroups.

Group A1, B1 (H file + GG): The coronal third was enlarged with GG drills #3 and #2 (Mani, INC., Tochigi, Japan) at 1500 rpm. The procedure continued using Hedstrom files (#30 to #20) in peripheral push-pull movements along the canal walls. After reaching the WL with a #15 K-file (Dentsply Maillefer, Ballaigues, Switzerland), the apical enlargement to size 25 was performed.

Group A2, B2 (PTUR): The retreatment procedure was carried out with a rotational speed of 500 rpm and a torque set at 2 Ncm. D1 (30/0.09), D2 (25/0.08), and D3 (20/0.07) were used sequentially in the coronal, middle and apical thirds, respectively, for gutta-percha removal.

Group A3, B3 (Rec): The R25 file (25/0.08) was used on the WL with three consecutive reciprocating pecking motions, then withdrawn for debris removal.

Group A4, B4 (MM Remover): This single-file system (30/0.07) was used at a speed of 500 rpm and a torque of 2.5 Ncm. The file was operated with a gentle brushing motion without applying apical pressure. When resistance was encountered, the file was withdrawn from the RC for debris removal.

All retreatment procedures, except for those performed with hand files, were conducted using the AI Motor T-mode Yoshi Terauchi Edition (Woodpecker), following the manufacturer’s protocol. Each rotary file was used only once per RC. After each removal, irrigation was performed using 2.5% NaOCl (2 mL). Retreatment was considered complete when no filling material remained on the instrument surface and the RC walls were free of detectable obturation material [7,21]. To standardize the procedure, the WL was reached five times in each canal [15]. Retreatment time was recorded from first instrument use, excluding irrigation and instrument-change time. Subsequently, the RCs were irrigated sequentially with 17% EDTA, 2.5% NaOCl, and distilled water, each in a volume of 2 mL. The RCs were dried with sterile paper points, and the coronal access cavities were sealed temporarily with Cavit-G. All retreatment procedures were carried out by the same clinician to ensure consistency.

### 2.4. Micro-CT Scanning, Reconstruction and Analysis

**Figure 1:**
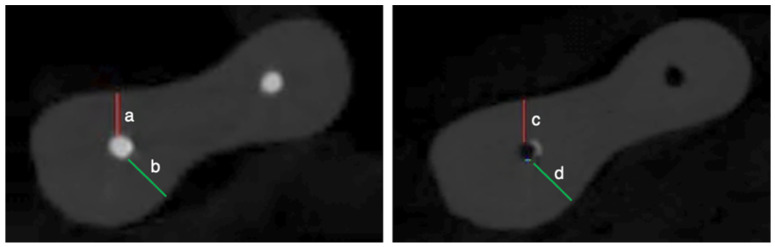
Micro-CT images showing the shortest distances measured between the root canal and the external root surface in the mesial (a: after obturation; c: after retreatment) and distal (b: after obturation; d: after retreatment) directions in a sample.

All specimens were scanned with a SkyScan 1275 micro-CT device (Bruker MicroCT, Kontich, Belgium) following both obturation and retreatment procedures. Scanning was performed at 80 kVp and 125 µA with a 360° rotation, a 0.2° rotational step, a pixel size of 15.0 μm, and a 1 mm aluminum filter. On average, 1800 cross-sectional slices were obtained per sample. 3D reconstruction was carried out using NRecon software (version 1.7.4.2, Bruker MicroCT, Kontich, Belgium) with predefined parameters based on a previous study [26]. Reconstructed cross-sections were transferred to CTAn software (version 1.23.0.2, Bruker MicroCT, Kontich, Belgium) for quantitative analysis, morphometric measurements, and 3D visualization. RC filling material was isolated and quantified through segmentation of volumes of interest following both obturation and retreatment. All volumetric measurements were expressed in cubic micrometers (μm^3^). The residual filling material volume for each RC was calculated as a percentage using the formula: (A/B) × 100, where A is the residual filling material volume and B is the initial obturation volume. Apical transportation was analyzed using DataViewer (version 1.5.6.2, Bruker Micro-CT, Kontich, Belgium). Axial sections located three mm coronal to the apical foramen were selected from post-obturation and post-retreatment images (Figure 1). Apical transportation (μm) was calculated using the published formula [7]. An apical transportation value of ‘0’ indicates no apical transportation; positive values indicate mesial transportation of the RC, whereas negative values indicate distal transportation. All images were analyzed and measured twice by the same observer with over 10 years of experience in micro-CT evaluation, with a one-month interval between assessments, to evaluate intra-observer repeatability using the intraclass correlation coefficient (ICC).

### 2.5. Statistical Analysis

Data normality was evaluated with the Shapiro–Wilk test. The Kruskal–Wallis H test was applied to compare the groups, since the data for residual filling material percentage and retreatment time were not distributed normally. When a statistically significant difference was detected among more than two groups, pairwise comparisons were performed using the Bonferroni-corrected Mann–Whitney U test. A One-Way ANOVA was conducted to compare the data for apical transportation, as it exhibited a normal distribution. Intra-observer repeatability was evaluated using the ICC, according to commonly accepted thresholds [27]. Analyses were performed in IBM SPSS Statistics (version 11.5, SPSS Inc., Chicago, IL, USA), and *p* < 0.05 was accepted as statistically significant.

## 3. Results

The mean retreatment times (in seconds) obtained for each file system used during the retreatment procedure are presented in Table 1. Statistical analysis revealed a significant difference among the groups (*p* < 0.001). Group B1 (CWC–H file + GG) exhibited a significantly longer mean retreatment time than that of Group A2 (SC–PTUR) (*p* = 0.044), Group A4 (SC–MM Remover) (*p* < 0.001), Group B2 (CWC–PTUR) (*p* = 0.001), and Group B4 (CWC–MM Remover) (*p* = 0.005) (*p* < 0.05).

**Figure 2:**
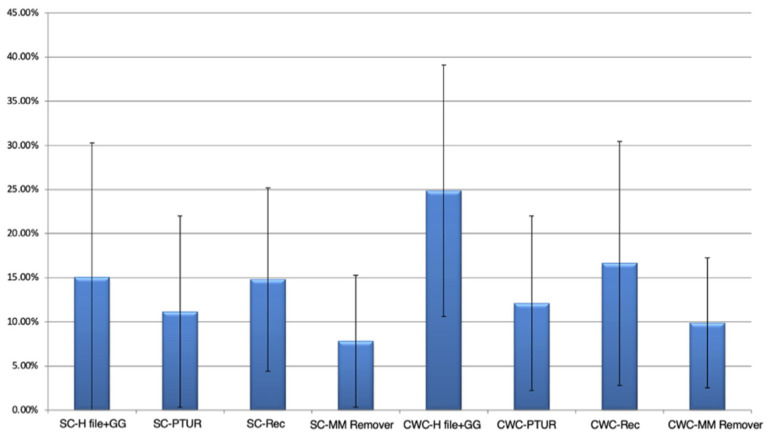
The mean percentages and standard deviations of the residual root canal filling materials after retreatment for each group.

**Figure 3:**
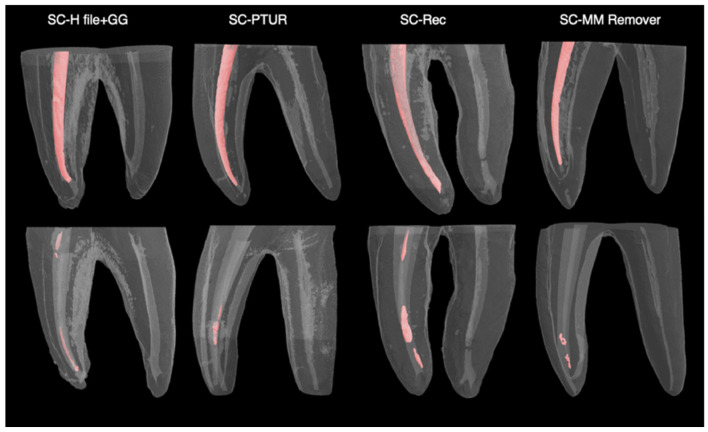
The three-dimensional micro-CT images of the groups obturated using the single-cone technique after root canal obturation and retreatment procedure.

**Figure 4:**
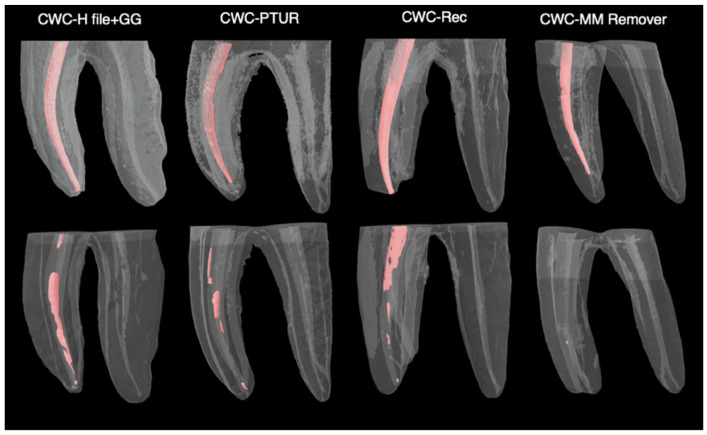
The three-dimensional micro-CT images of the groups obturated using the continuous wave vertical compaction technique after root canal obturation and retreatment procedure.

The mean percentages of residual RC filling material for each group are presented in Table 2 and Figure 2. The lowest mean percentage of residual filling material was observed in Group A4 (SC–MM Remover), while the highest was recorded in Group B1 (CWC–H file + GG). However, Kruskal–Wallis H test revealed no statistically significant differences among the groups regarding the residual filling material (%) (*p* > 0.05). Micro-CT images of the groups obturated using the SC (Figure 3) and CWC techniques (Figure 4), following RC obturation and retreatment, are also presented.

The apical transportation values (µm) of the experimental groups are shown in Table 3, with negative values observed in Groups A1 (SC–H file + GG), A2 (SC–PTUR), A3 (SC–Rec), and B3 (CWC–Rec), whereas positive values were recorded in Groups A4 (SC–MM Remover), B1 (CWC–H file + GG), B2 (CWC–PTUR), and B4 (CWC–MM Remover). The highest mean apical transportation in the distal direction was observed in Group B3 (−108.57 µm), while the highest in the mesial direction was found in Group B2 (+154.29 µm). However, according to the statistical evaluation, none of the differences among the groups were statistically significant (*p* > 0.05). Intra-observer repeatability demonstrated good to excellent reliability, with ICCs ranging from 0.87 to 0.90 across the evaluated measurements.

## 4. Discussion

When RC treatment fails, nonsurgical retreatment is often indicated. Instrument performance plays a crucial role in filling material removal during retreatment procedures, especially in curved RCs [28]. Previous studies have shown that none of the existing techniques can completely eliminate filling materials from the RC system [6,7,29]. This circumstance has led to a continuous search for an ideal technique or material.

Mandibular molars often present mesial roots with complex morphology and frequent curvatures [30,31]. Owing to their challenging anatomy, these teeth were selected for inclusion in the present study.

Currently, the CWC technique is widely preferred because it produces a more homogeneous volume of gutta-percha and better adaptation to RC curvatures [32,33]. However, warm obturation techniques may make the removal of RC filling materials more challenging when retreatment is required [34]. Despite various studies on retreatment efficacy of different instruments in RCs filled with warm or cold techniques, comparative data for curved RCs comparing warm versus cold obturation remain limited.

Various methods have been used to assess residual filling materials and apical transportation following retreatment, including two-dimensional (2D) radiographic examination [9,14,35], image analyzer software [36,37], and 3D tomographic techniques such as cone-beam computed tomography (CBCT) [20,38] and micro-CT [7,15]. Micro-CT offers higher resolution images with minimal artifacts compared to other imaging techniques [39,40]. These advantages enable accurate 3D visualization and quantitative assessment of RC morphology, dentinal removal and residual filling material following retreatment [10]. Therefore, it was selected as the imaging technique in the present study.

In this study, the retreatment effectiveness of PTUR, Rec, Hedstrom files, and the newly introduced MM Remover was evaluated with respect to residual filling material volume, apical transportation, and retreatment time in curved RCs. In this context, the evaluated instruments exhibit variability in metallurgical properties and design characteristics with respect to cross-sectional geometry, alloy composition, tip configuration, and taper design. The Rec system features an S-shaped cross-sectional design [7] and is manufactured using M-wire alloy [34]. By comparison, PTUR presents a triangular cross-sectional design [20], while the MM Remover is produced with a C-wire alloy and incorporates a variable triple-helix cross-sectional geometry. Therefore, these differences are challenging to standardize and may influence the retreatment performance of instruments in curved RCs.

According to the findings of the study, although differences in residual filling material volumes were observed among the groups, these differences were not statistically significant (*p* > 0.05). This finding aligns with prior research by Rödig et al. [6] and Adel et al. [20], who also reported no significant differences between PTUR, Rec, and Hedstrom instruments in removing RC filling materials from curved RCs. However, Serefoglu et al. [14] reported that the Rec left more residual filling material than Hedstrom files in retreatment procedures of curved RCs obturated with the SC technique. The researchers utilized a 2D imaging method for evaluation in their studies, whereas the use of the more accurate 3D micro-CT in our research can explain the difference in the results. Bago et al. [15] evaluated the PTUR, Rec, Rec Blue, and Wave One Gold file systems in removing RC filling materials from curved RCs obturated using the CWC technique and reported no significant differences, which is consistent with our findings regarding PTUR and Rec. Within this context, the lowest mean volume of residual filling material was observed in the group using the MM Remover file system in RCs filled with the SC technique, whereas the highest mean volume was found in the Hedstrom hand file group in RCs filled with the CWC technique. Although the MM Remover file system left the least amount of residual filling material in both obturation techniques, the difference was not statistically significant (*p* > 0.05). However, this observation may be related to its variable triple-helix cross-section design, which could contribute to improved cutting efficiency and debris removal in curved RCs.

Studies have shown that solvents soften gutta-percha, making it more viscous and adhesive, which may lead to its penetration into canal irregularities or dentinal tubules [41,42,43]. Such alterations in the physical properties of the filling material may complicate the retreatment procedure, making it more time-consuming or technically challenging [44]. Therefore, in the present study, the use of solvents was deliberately avoided during the retreatment procedures.

Although apical transportation in both mesial and distal directions was observed for all instruments, no statistically significant differences were found among the groups in terms of mean transportation values (*p* > 0.05). This finding is consistent with the results reported by Da Silva Arruda et al. [38], who found no significant differences among PTUR, ProTaper Next, and Rec in curved RCs. Similarly, Adel et al. [20] observed that PTUR, Rec, and Hedstrom hand files showed similar apical transportation outcomes in the apical 3 mm section of curved RCs. In this context, the comparable apical transportation exhibited by the MM Remover relative to the other file systems may be attributed to its heat-treated technology, which promotes controlled RC wall contact and limits excessive dentin removal.

Apical transportation greater than 0.3 mm has been associated with reduced apical sealing capacity of RC filling materials, which may negatively impact the clinical outcome of endodontic treatment [45,46,47]. In the present study, the mean apical transportation values of all tested systems remained below this critical threshold, indicating that the instruments were able to perform retreatment within clinically acceptable limits without compromising the sealing ability of RC filling materials or long-term endodontic treatment success, while maintaining the original canal morphology.

With respect to canal curvature, positive apical transportation values reflect mesial deviation toward the outer wall of the RC, whereas negative values represent distal deviation toward the inner wall [46]. In curved RCs, rotary instruments may lead to increased apical dentin removal along the outer curvature [48], and the relatively higher mesial apical transportation values observed in some PTUR specimens may be attributed to the inherent tendency of instruments to straighten, resulting in deviation toward the outer canal wall [47,49].

From a kinematic perspective, the tested systems differed in their mode of motion, with the PTUR and MM Remover being operated in continuous rotary motion, the Rec being used in reciprocating motion, and Hedstrom files being applied with manual instrumentation. These differences in kinematics may influence the observed transportation patterns in curved RCs. In light of these findings and within the limitations of the present study, the lack of detectable differences in apical transportation may be attributable to the limited sample size, potentially increasing the risk of a type II error. Expanding the sample size in future investigations may improve the detection of differences.

In our study, PTUR and MM Remover achieved shorter retreatment times than the other systems, regardless of obturation technique. The CWC-H file + GG group exhibited significantly longer retreatment times (*p* < 0.05). These results are consistent with previous studies [6,20], which reported similar retreatment times for PTUR and Rec, but longer durations with Hedstrom files. Similarly, another study reported significantly longer retreatment times for the Hedstrom group compared with PTUR and Rec; however, unlike our findings, PTUR required more time than Rec [23]. MM Remover’s reduced retreatment time may be attributed to its triple-helix cross-sectional design and thermomechanical processing [16]. From a clinical perspective, reduced retreatment time may offer important advantages by improving patient comfort, decreasing operator fatigue, and enhancing procedural efficiency. In addition, previous clinical studies have demonstrated the success of single-visit retreatment [1,50]; therefore, shorter procedures may increase the feasibility of completing retreatment in a single appointment.

In this study, the criterion for retreatment completion, defined as the absence of filling material on the final instrument surface, may be operator-dependent; however, this protocol was applied in line with previously published retreatment studies [7,15,21]. Moreover, the use of micro-CT provided an objective and quantitative assessment of residual filling material, thereby minimizing observer-related subjectivity.

The use of Gates–Glidden drills was limited to the Hedstrom file group to reflect conventional clinical practice with hand instrumentation and to facilitate coronal flaring [6,20]. However, rotary retreatment systems are generally designed to achieve coronal enlargement through their instrument design and kinematics; therefore, auxiliary coronal flaring drills were not used in the present study [23].

Despite the noteworthy findings of the current research, achieving complete cleanliness of the RC system during non-surgical retreatment remains a persistent challenge. This research was conducted under in vitro conditions using mandibular molars with curved mesial RCs, which may not fully reflect clinical variability. Anatomical differences such as root curvature and canal morphology may influence retreatment efficacy and limit the generalizability of the results. To the best of our knowledge, no previous study has evaluated the retreatment efficacy of the MM Remover system in curved RCs; therefore, the present study was designed to compare the performance of this system with commonly used rotary, reciprocating, and traditional hand file systems under conditions that reflect their typical clinical application. The results obtained in controlled in vitro conditions not only clarify whether the anticipated advantages of the system—such as improved efficiency, enhanced safety, and avoidance of solvents—are realized in practice, but also offer valuable insights into its performance within anatomically complex scenarios, particularly in the mesial roots of mandibular molars. These findings enrich the current evidence base and contribute to the ongoing efforts to optimize retreatment protocols for challenging endodontic cases. Continued development of more efficient file systems tailored to complex RC anatomies is essential to improve outcomes. Future studies should include larger sample sizes, broader clinical trials, and assessment of adjunctive irrigation activation to enhance filling material removal.

## 5. Conclusions

Considering the constraints of this study, all evaluated file systems were able to remove filling materials from curved RCs effectively while preserving the original canal anatomy and avoiding excessive apical transportation. Although the MM Remover system left the lowest residual filling material, the differences among all file systems regarding filling material removal and apical transportation were not statistically significant. Nevertheless, both the PTUR and MM Remover systems showed significantly shorter retreatment times, indicating clinical advantages in terms of efficiency. These findings highlight the potential clinical applicability of the newly developed MM Remover, supporting its further evaluation in anatomically complex cases, particularly in the curved mesial roots of mandibular molars.

## Figures and Tables

**Table 1:** The minimum, maximum, median, and mean ± standard deviation values (in seconds) of the retreatment times for each group.

Group	Mean ± SD (s)	Median (s)	Minimum (s)	Maximum (s)	*p* Value
A1 (SC–H file + GG)	508.00 ± 146.67	570.00	241.00	663.00	*p* < 0.001 *
A2 (SC–PTUR)	327.43 ± 88.32	322.00	191.00	465.00	
A3 (SC–Rec)	352.29 ± 52.26	354.00	295.00	444.00	
A4 (SC–MM Remover)	254.43 ± 57.07	235.00	185.00	317.00	
B1 (CWC–H-file + GG)	708.29 ± 69.01	725.00	597.00	792.00	
B2 (CWC–PTUR)	300.00 ± 98.43	265.00	176.00	438.00	
B3 (CWC–Rec)	461.00 ± 171.45	510.00	265.00	695.00	
B4 (CWC–MM Remover)	299.43 ± 34.90	298.00	268.00	370.00	

Kruskal–Wallis H test * (*p* < 0.05); (n = 32 per main group, n = 8 per subgroup).

**Table 2:** The minimum, maximum, median, and mean ± standard deviation values (%) of residual root canal filling material for each group.

Group	Mean ± SD (%)	Median (%)	Minimum (%)	Maximum (%)	*p* Value
A1 (SC–H file + GG)	15.05 ± 15.23	11.83	0.00	41.69	*p* = 0.339 *
A2 (SC–PTUR)	11.17 ± 10.82	6.48	0.78	26.79	
A3 (SC–Rec)	14.80 ± 10.39	13.54	2.19	34.45	
A4 (SC–MM Remover)	7.81 ± 7.48	3.78	1.45	22.18	
B1 (CWC–H file + GG)	24.86 ± 14.34	22.77	3.03	48.50	
B2 (CWC–PTUR)	12.09 ± 9.89	15.57	0.49	22.67	
B3 (CWC–Rec)	16.63 ± 13.82	11.28	1.87	41.82	
B4 (CWC–MM Remover)	9.88 ± 7.35	10.04	1.11	24.55	

Kruskal–Wallis H test * (*p* > 0.05); (n = 32 per main group, n = 8 per subgroup).

**Table 3:** The minimum, maximum, median, and mean ± standard deviation values (µm) of apical transportation for each group.

Group	Mean ± SD	Median	Minimum	Maximum	*p* Value
A1 (SC–H file + GG)	−17.14 ± 166.30	−80.00	−240.00	200.00	*p* = 0.073 *
A2 (SC–PTUR)	−34.29 ± 114.14	−40.00	−160.00	80.00	
A3 (SC–Rec)	−17.14 ± 134.38	0.00	−200.00	160.00	
A4 (SC–MM Remover)	62.86 ± 145.80	40.00	−120.00	280.00	
B1 (CWC–H file + GG)	34.29 ± 111.78	40.00	−120.00	200.00	
B2 (CWC–PTUR)	154.29 ± 201.90	80.00	0.00	480.00	
B3 (CWC–Rec)	−108.57 ± 111.87	−100.00	−280.00	40.00	
B4 (CWC–MM Remover)	71.43 ± 181.79	100.00	−280.00	240.00	

One-Way ANOVA test * (*p* > 0.05); (n = 32 per main group, n = 8 per subgroup).

## Data Availability

The original contributions presented in this study are included in the article. Further inquiries can be directed to the corresponding author.

## Abbreviations

RC	Root Canal
NiTi	Nickel–Titanium
3D	Three-dimensional
Micro-CT	Micro-Computed Tomography
WL	Working Length
NaOCl	Sodium Hypochlorite
EDTA	Ethylenediaminetetraacetic Acid
ICC	Intraclass Correlation Test
2D	Two-dimensional

## References

[1] Toia C.C., Khoury R.D., Corazza B.J.M., Orozco E.I.F., Valera M.C. (2022). Effectiveness of 1-Visit and 2-Visit Endodontic Retreatment of Teeth with Persistent/Secondary Endodontic Infection: A Randomized Clinical Trial with 18 Months of Follow-up. J. Endod..

[2] Song M., Kim H.C., Lee W., Kim E. (2011). Analysis of the cause of failure in nonsurgical endodontic treatment by microscopic inspection during endodontic microsurgery. J. Endod..

[3] Tabassum S., Khan F.R. (2016). Failure of endodontic treatment: The usual suspects. Eur. J. Dent..

[4] Marques da Silva B., Baratto-Filho F., Leonardi D., Henrique Borges A., Volpato L., Branco Barletta F. (2012). Effectiveness of ProTaper, D-RaCe, and Mtwo retreatment files with and without supplementary instruments in the removal of root canal filling material. Int. Endod. J..

[5] Alves F.R., Ribeiro T.O., Moreno J.O., Lopes H.P. (2014). Comparison of the efficacy of nickel-titanium rotary systems with or without the retreatment instruments in the removal of gutta-percha in the apical third. BMC Oral Health.

[6] Rödig T., Reicherts P., Konietschke F., Dullin C., Hahn W., Hülsmann M. (2014). Efficacy of reciprocating and rotary NiTi instruments for retreatment of curved root canals assessed by micro-CT. Int. Endod. J..

[7] Nevares G., de Albuquerque D.S., Freire L.G., Romeiro K., Fogel H.M., Dos Santos M., Cunha R.S. (2016). Efficacy of ProTaper Next compared with Reciproc in removing obturation material from severely curved root canals: A micro-computed tomography study. J. Endod..

[8] Hülsmann M., Stotz S. (1997). Efficacy, cleaning ability and safety of different devices for gutta-percha removal in root canal retreatment. Int. Endod. J..

[9] Aguiar C.M., Lima G.D.C., Bernart F.D., Câmara A.C. (2011). Effectiveness of the ProTaper Universal Retreatment™ System and the manual technique in endodontic retreatment. Acta Stomatol. Croat..

[10] Rödig T., Hausdörfer T., Konietschke F., Dullin C., Hahn W., Hülsmann M. (2012). Efficacy of D-RaCe and ProTaper Universal Retreatment NiTi instruments and hand files in removing gutta-percha from curved root canals–a micro-computed tomography study. Int. Endod. J..

[11] Ozyurek T., Ozsezer-Demiryurek E. (2017). Efficacy of ProTaper Next and ProTaper Universal retreatment systems in removing gutta-percha in curved root canals during root canal retreatment. J. Istanb. Univ. Fac. Dent..

[12] Metzger Z., Ben-Amar A. (1995). Removal of overextended gutta-percha root canal fillings in endodontic failure cases. J. Endod..

[13] Ersev H., Yılmaz B., Dinçol M., Dağlaroğlu R. (2012). The efficacy of ProTaper Universal rotary retreatment instrumentation to remove single gutta-percha cones cemented with several endodontic sealers. Int. Endod. J..

[14] Serefoglu B., Kandemir Demirci G., Micoogullari Kurt S., Kasikci Bilgi I., Caliskan M.K. (2021). Impact of root canal curvature and instrument type on the amount of extruded debris during retreatment. Restor. Dent. Endod..

[15] Bago I., Plotino G., Katic M., Rocan M., Batinic M., Anic I. (2020). Evaluation of filling material remnants after basic preparation, apical enlargement and final irrigation in retreatment of severely curved root canals in extracted teeth. Int. Endod. J..

[16] Özkan H.B., Sürme K., Akman H., Er K. (2023). Comparison of cyclic fatigue resistance between heat-treated and conventional retreatment files. Rev. Port. Estomatol. Med. Dent. Cir. Maxilofac..

[17] Coltene: MicroMega Remover. https://media.coltene.com/EN/GB/media/DOC_FLY_60301861-A-06-20-EN-MicroMega-REMOVER-A4_IND.pdf?sprache=EN.

[18] Kırıcı D., Demirbuga S., Karatas E. (2020). Micro-computed tomographic assessment of the residual filling volume, apical transportation, and crack formation after retreatment with Reciproc and Reciproc Blue systems in curved root canals. J. Endod..

[19] Liang A., Huang L., Li B., Huang Y., Zhou X., Zhang X., Gong Q. (2022). Micro-CT evaluation of different root canal irrigation protocols on the removal of accumulated hard tissue debris: A systematic review and meta-analysis. J. Clin. Med..

[20] Adel M., Tofangchiha M., Rashvand E., Moutabha I., Roohi N., Reda R., Testarelli L. (2022). Comparison of the efficacy of NeoNiTi, ProTaper, and Reciproc files in the retreatment of curved root canals: A CBCT assessment. Acta Stomatol. Croat..

[21] Bernardes R.A., Duarte M.A.H., Vivan R.R., Alcalde M.P., Vasconcelos B.C., Bramante C.M. (2016). Comparison of three retreatment techniques with ultrasonic activation in flattened canals using micro-computed tomography and scanning electron microscopy. Int. Endod. J..

[22] Pop I., Manoharan A., Zanini F., Tromba G., Patel S., Foschi F. (2015). Synchrotron light-based μCT to analyse the presence of dentinal microcracks post-rotary and reciprocating NiTi instrumentation. Clin. Oral Investig..

[23] Orhan E., Orhan K., Ocak M., Gunes B. (2025). Comparing the root canal filling removal efficiency of XP-endo retreatment system with hand Files, R-endo, reciproc and protaper universal retreatment systems in curved root canals: A micro-CT study. BMC Oral Health.

[24] Schneider S.W. (1971). A comparison of canal preparations in straight and curved root canals. Oral Surg. Oral Med. Oral Pathol..

[25] Vertucci F.J. (1984). Root canal anatomy of the human permanent teeth. Oral Surg. Oral Med. Oral Pathol..

[26] Odabaşı Tezer E., Kırmızı D., Abduljalil M., Basmacı F., Buyuksungur A., Dartar Öztan M. (2024). Comparative retreatment efficacy of two Multi-file systems with different Access cavity designs: A Micro-computed Tomography Study. Medicina.

[27] Koo T.K., Li M.Y. (2016). A guideline of selecting and reporting intraclass correlation coefficients for reliability research. J. Chiropr. Med..

[28] Schirrmeister J.F., Wrbas K.T., Schneider F.H., Altenburger M.J., Hellwig E. (2006). Effectiveness of a hand file and three nickel-titanium rotary instruments for removing gutta-percha in curved root canals during retreatment. Oral Surg. Oral Med. Oral Pathol. Oral Radiol. Endod..

[29] Alves F.R.F., Marceliano-Alves M.F., Sousa J.C.N., Silveira S.B., Provenzano J.C., Siqueira J.F. (2016). Removal of root canal fillings in curved canals using either reciprocating single- or rotary multi-instrument systems and a supplementary step with the XP-Endo Finisher. J. Endod..

[30] de Pablo O.V., Estevez R., Peix Sanchez M., Heilborn C., Cohenca N. (2010). Root anatomy and canal configuration of the permanent mandibular first molar: A systematic review. J. Endod..

[31] Kartal N., Cimilli H.K. (1997). The degrees and configurations of mesial canal curvatures of mandibular first molars. J. Endod..

[32] Fracassi L.D., Ferraz E.G., Albergaria S.J., Veeck E.B., da Costa N.P., Sarmento V.A. (2013). Evaluation of the quality of different endodontic obturation techniques by digital radiography. Clin. Oral Investig..

[33] Santos-Junior A.O., Tanomaru-Filho M., Pinto J.C., Tavares K., Torres F.F.E., Guerreiro-Tanomaru J.M. (2021). Effect of obturation technique using a new bioceramic sealer on the presence of voids in flattened root canals. Braz. Oral Res..

[34] Romeiro K., de Almeida A., Cassimiro M., Gominho L., Dantas E., Chagas N., Velozo C., Freire L., Albuquerque D. (2020). Reciproc and Reciproc Blue in the removal of bioceramic and resin-based sealers in retreatment procedures. Clin. Oral Investig..

[35] Çelik Ünal G., Üreyen Kaya B., Taç A., Keçeci A. (2009). A comparison of the efficacy of conventional and new retreatment instruments to remove gutta-percha in curved root canals: An ex vivo study. Int. Endod. J..

[36] Zuolo A.S., Mello J.E., Cunha R.S., Zuolo M.L., Bueno C.E. (2013). Efficacy of reciprocating and rotary techniques for removing filling material during root canal retreatment. Int. Endod. J..

[37] de Oliveira D.P., Barbizam J.V.B., Trope M., Teixeira F.B. (2006). Comparison between gutta-percha and resilon removal using two different techniques in endodontic retreatment. J. Endod..

[38] da Silva Arruda E., Sponchiado-Júnior E.C., Pandolfo M.T., de Carvalho Fredson M.A., Garcia L.d.F.R., Marques A.A.F. (2019). Apical transportation and centering ability after root canal filling removal using reciprocating and continuous rotary systems: A CBCT study. Eur. J. Dent..

[39] Fischer B.V., Goulart T.S., Vitali F.C., de Souza D.L., da Silveira Teixeira C., Garcia L.d.F.R. (2024). Supplementary methods for filling material removal: A systematic review and meta-analysis of micro-CT imaging studies. J. Dent..

[40] Faus-Matoses V., Pasarín-Linares C., Faus-Matoses I., Foschi F., Sauro S., Faus-Llácer V.J. (2020). Comparison of obturation removal efficiency from straight root canals with protaper gold or reciproc blue: A micro-computed tomography study. J. Clin. Med..

[41] Bhagavaldas M.C., Diwan A., Kusumvalli S., Pasha S., Devale M., Chava D.C. (2017). Efficacy of two rotary retreatment systems in removing gutta-percha and sealer during endodontic retreatment with or without solvent: A comparative in vitro study. J. Conserv. Dent. Endod..

[42] Latheef A.A., Miglani R., Indira R., Kader M.A., Nasim V.S., Shamsuddin S.V. (2016). Effect of passive ultrasonic irrigation on the cleanliness of dentinal tubules in non-surgical endodontic retreatment with and without solvent: A scanning electron microscope study. J. Int. Oral Health.

[43] Ma J., Al-Ashaw A.J., Shen Y., Gao Y., Yang Y., Zhang C., Haapasalo M. (2012). Efficacy of ProTaper Universal rotary retreatment system for gutta-percha removal from oval root canals: A micro-computed tomography study. J. Endod..

[44] Rossi-Fedele G., Ahmed H.M. (2017). Assessment of Root Canal Filling Removal Effectiveness Using Micro-computed Tomography: A Systematic Review. J. Endod..

[45] Wu M.K., Fan B., Wesselink P.R. (2000). Leakage along apical root fillings in curved root canals. Part I: Effects of apical transportation on seal of root fillings. J. Endod..

[46] Romeo V.R., La Rosa G.R.M., Generali L., Pedullà E., Angerame D. (2025). Influence of different kinematics on shaping ability and accumulated hard tissue debris: An ex vivo study. Odontology.

[47] Razcha C., Zacharopoulos A., Anestis D., Mikrogeorgis G., Zacharakis G., Lyroudia K. (2020). Micro-computed tomographic evaluation of canal transportation and centering ability of 4 heat-treated nickel-titanium systems. J. Endod..

[48] Puleio F., Bellezza U., Torre A., Giordano F., Lo Giudice G. (2023). Apical transportation of apical foramen by different NiTi alloy systems: A systematic review. Appl. Sci..

[49] Junaid A., Freire L.G., da Silveira Bueno C.E., Mello I., Cunha R.S. (2014). Influence of single-file endodontics on apical transportation in curved root canals: An ex vivo micro–computed tomographic study. J. Endod..

[50] Eyuboglu T.F., Olcay K., Özcan M. (2017). A clinical study on single-visit root canal retreatments on consecutive 173 patients: Frequency of periapical complications and clinical success rate. Clin. Oral Investig..

